# The Italian national survey on Coronavirus disease 2019 epidemic spread in nursing homes

**DOI:** 10.1002/gps.5487

**Published:** 2021-01-02

**Authors:** Flavia L. Lombardo, Ilaria Bacigalupo, Emanuela Salvi, Eleonora Lacorte, Paola Piscopo, Flavia Mayer, Antonio Ancidoni, Giulia Remoli, Guido Bellomo, Gilda Losito, Fortunato D'Ancona, Antonio Bella, Patrizio Pezzotti, Marco Canevelli, Graziano Onder, Nicola Vanacore, the Italian National Institute of Health Nursing Home Study Group

**Affiliations:** ^1^ National Center for Disease Prevention and Health Promotion Italian National Institute of Health Rome Italy; ^2^ National Center for Drug Research and Evaluation Italian National Institute of Health Rome Italy; ^3^ Department of Neuroscience Italian National Institute of Health Rome Italy; ^4^ Department of Human Neuroscience Sapienza University of Rome Rome Italy; ^5^ Italian National Guarantor for the Rights of Persons Detained or Deprived of Liberty Rome Italy; ^6^ Department of Infectious Diseases Italian National Institute of Health Rome Italy; ^7^ Department of Cardiovascular Endocrine‐metabolic Diseases and Aging, Italian National Institute of Health Rome Italy

**Keywords:** COVID‐19, nursinghome, public health

## Abstract

**Introduction:**

Residents in facilities such as nursing homes (NHs) are particularly vulnerable to Coronavirus disease 2019 (COVID‐19). A national survey was carried out to collect information on the spreading and impact of severe acute respiratory syndrome coronavirus 2 (SARS‐CoV‐2) infection in nursing homes, and on how suspected and/or confirmed cases were managed. We carried out a survey between 25 March 2020 and 5 May 2020.

**Materials and Methods:**

All Italian nursing homes either public or providing services both privately and within the NHS were included in the study. An on‐line questionnaire was sent to 3292 nursing homes across all Italian regions. Nursing homes were also contacted by telephone to provide assistance in completing the questionnaire.

**Results:**

A total of 1356 nursing homes voluntarily participated to the survey, hosting a total of 100,806 residents. Overall, 9154 residents died due to any cause from February 1 to the time when the questionnaire was completed (from March 25 to May 5). Of these, 7.4% had COVID‐19 and 33.8% had flu‐like symptoms, corresponding to a cumulative incidence of 0.7 and 3.1, respectively. Lack of personnel, difficulty in transferring patients to hospital or other facility, isolating residents with COVID‐19, number of beds and geographical area were the main factor positively associated to the presence of COVID‐19 in nursing homes.

**Discussion:**

This survey showed the dissemination and impact of SARS‐CoV‐2 infection in Italian nursing homes and on how older and potentially chronically ill people residing in these long‐term care facilities were managed.

## Key points

- Dissemination and impact of severe acute respiratory syndrome coronavirus 2 infection in Italian nursing homes
- How elderly and potentially chronically ill people residing in these long‐term care facilities were managed
- The mortality profile in the Italian nursing homes

## 1 INTRODUCTION

As of July 26, a total of 16,048,100 cases of infection with severe acute respiratory syndrome coronavirus 2 (SARS‐CoV‐2) were reported worldwide, and 644,537 deaths, with the United States (4,178,027 cases and 146,460 deaths), Brazil (2,394,513 cases and 86,449 deaths), and India (1,385,635 cases and 32,060 deaths) being the countries that have currently reported the higher number of cases.^1^

Italy reported 245,864 cases and 35,102 deaths.^2^, ^3^, ^4^

Coronavirus disease 2019 (COVID‐19), resulting from SARS‐CoV‐2 infection, is an acute respiratory infection mainly involving the lower respiratory tract. Symptoms are usually mild, and some people may also remain completely asymptomatic throughout the course of the disease. However, some people can develop more severe symptoms, that may lead to life threatening complications and even death. This unfavorable course has mostly been observed among frail, older people, who are paying the highest toll in the ongoing pandemic.^5^ An analysis of a subgroup of patients with COVID‐19 deceased in Italy confirmed the higher mean age of patients (79.5 years) and the higher frequency of underlying conditions such as ischemic heart disease (30%), diabetes (35.5%), active cancer (20.3%), or atrial fibrillation (24.5%), with 48.5% having ≥3 underlying conditions, with a case fatality rate of 12.8% in people aged 70–79 years and of 20.8% in people aged ≥80 years.^5^ Then, advancing age and the presence of concomitant chronic conditions (e.g., ischemic heart disease, diabetes, and cancer) have been reported as relevant risk factors for poorer outcomes.^5^

Therefore, the elderly and chronically ill people residing in long‐term care facilities such as nursing homes (NHs) are particularly vulnerable to COVID‐19^6^, ^7^, ^8^, ^9^, ^10^, ^11^, ^12^, ^13^ as they live in a communal setting along with other at‐risk people. This close contact^10^ ^,^ ^14^ can exponentially increase the risk of outbreaks of COVID‐19 within these structures.^7^ ^,^ ^9^ ^,^ ^10^ In addition, staff members in NHs often work in multiple facilities, including hospitals and clinics, thus increasing the risk of spreading the virus. This close contact, along with a higher vulnerability of older residents, due to their comorbid chronic conditions,^10^ ^,^ ^14^ can exponentially increase the risk of outbreaks of COVID‐19 within this type of facilities.^7^ ^,^ ^9^ ^,^ ^10^ Moreover, a lack of personal protective equipment (PPE) was recorded in all the country due to the sudden increase in the request, and facilities might not guarantee a timely isolation, transferral, and care of positive patients.^11^ The role of staff members in containing the infection is essential in both recognizing the symptoms among frail residents, and preventing and controlling the epidemic within the facility.^15^ All these aspects highlight the vulnerability of long‐term care facilities to COVID‐19 outbreaks and thus the need to protect and monitor the safety of both residents and staff members in NHs and other long‐term care facilities.^9^, ^10^, ^11^^,^ 16, 17, 18

In this context, the Italian National Institute of Health (INIH) in collaboration with the Italian National Guarantor for the rights of persons detained or deprived of liberty, carried out a flash survey aiming at collecting information, provided on a voluntary basis, on the spreading and impact of SARS‐CoV‐2 infection in NHs and on how potential cases were managed.

## 2 METHODS

**FIGURE 1:**
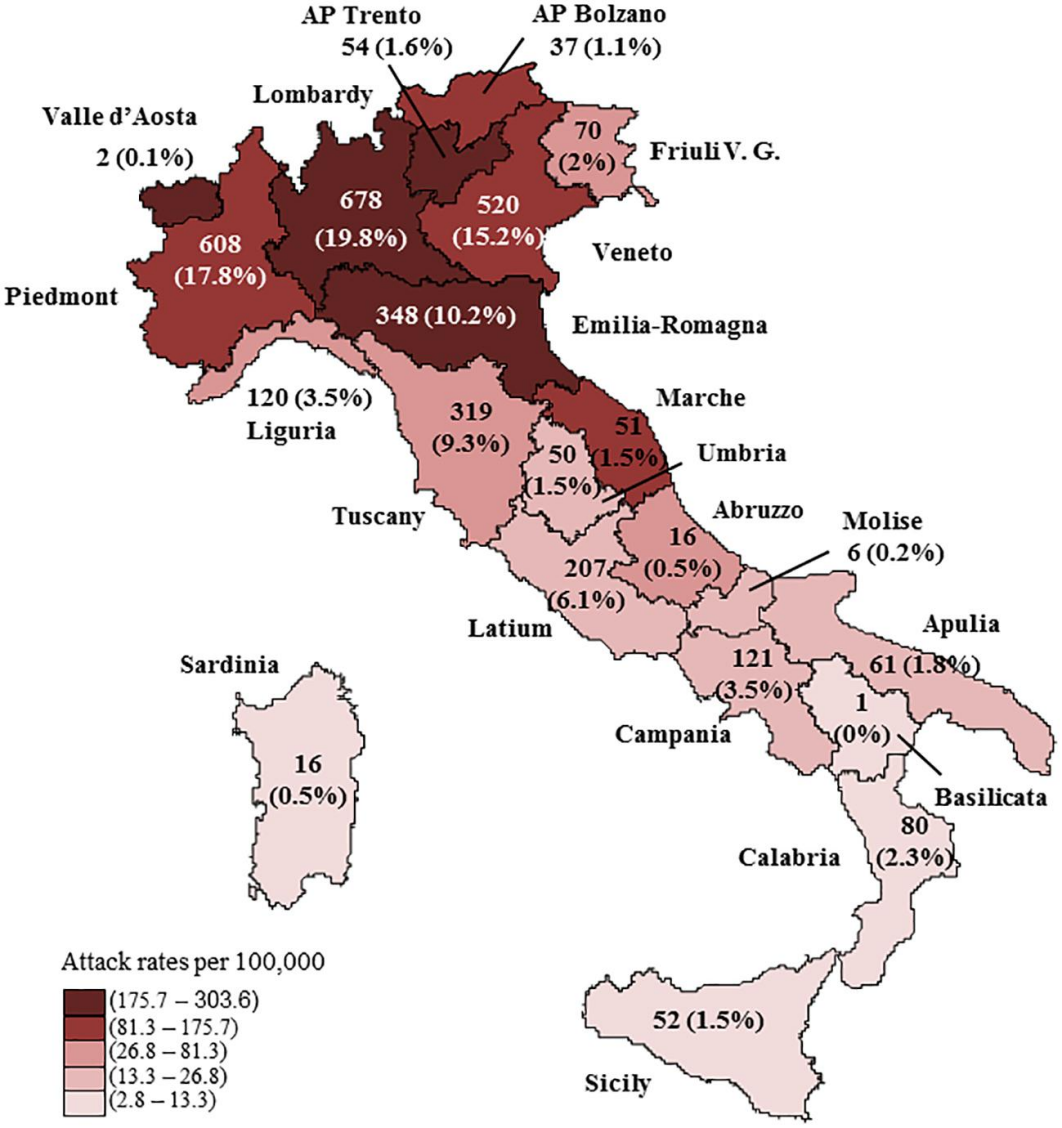
Number of nursing homes (percentage on the total) and COVID‐19 attack rates (per 100,000 habitants)

This national survey involved 3292 NHs, either public or providing services both privately and within the national health system, out of the 3417 NHs covering the whole Italian territory (Figure 1). We included all the NHs for which we had an available reference contact. The list of NHs was provided by the Dementia Observatory, an online map of Italian dementia services, constituting one of the objectives of the implementation of the Italian National Dementia Plan.^19^ ^,^ ^20^

### 2.1 Data source

A questionnaire with a cover letter was addressed to the director of each NH between 24 March and 27 April 2020. NHs were also contacted by telephone to provide assistance in completing the questionnaire. Some of the NHs were further contacted to solve incongruences in some of the provided data. The questionnaire was designed to gather information on:(1) the characteristics of the facility, including number of beds, type of structure (public or providing services both privately and within the NHS), number and type of healthcare and social workers (HCSW), residents living in the facilities; (2) the spreading of the infection, including the number of residents who died due to any cause in the considered time periods, and those who were SARS‐CoV‐2 positive or had flu‐like symptoms, the number of hospitalizations within the considered time period, and number of residents and staff members who had a SARS‐CoV‐2 positive test or flu‐like symptoms when the questionnaire was completed; and (3) the infection prevention and control (IPC) program components and practices that were adopted to manage patients with suspected or confirmed COVID‐19. Moreover, the questionnaire included a question on potential difficulties faced during the pandemic: (4) issues potentially related to the epidemic, including physical restraint measures, increase in the use of psychopharmacological drugs, and adverse events (data not shown). No information on individual residents and staff members were collected (see questionnaire in Supporting Information 1).

On 27 February 2020, the Italian Presidency of the Council of Ministers authorized the collection and scientific dissemination of data concerning the COVID‐19 epidemics by the INIH and other public health institutions.^21^

### 2.2 Statistical analysis

Descriptive statistics were performed on overall data and by region. Frequencies were used to describe dichotomous variables, means and standard deviations were used for continuous variable, and median and range values for data with asymmetric distributions. The nonparametric Spearman rank coefficient was used to assess potential correlations between measures. Data from the national surveillance system^22^ were used to test for possible correlations with the spreading of COVID‐19 at a regional level. Missing data on number of residents were imputed using the number of beds. No other data were imputed. An univariate and a multivariate regression logistic model were performed to assess whether critical aspects and characteristics of the NHs, adjusted for geographical area, were associated to COVID‐19 outbreaks defined as the presence of laboratory‐confirmed cases among deceased and hospitalized residents or staff members, and among residents currently living in the facility.

Interaction of factors with the time of response categorized as either within 3 weeks or after 3 weeks from the start of the survey, was tested. This cut‐off was chosen because after week 3 an increased proportion of NHs with an outbreak was observed compared to the previous weeks. A separate multivariate model was performed for lack of laboratory tests as these data were gathered starting from April 8.

A sensitivity analysis was performed including also flu‐like symptoms within the definition of COVID‐19 outbreak.

All data analyses were performed using STATA software, version 14.2 (Stata Corp).

The data that support the findings of this study are available on request from the corresponding author. The data are not publicly available due to privacy or ethical restrictions.

## 3 RESULTS

### 3.1 Response rate

Overall, 1356 (41.2%) of the 3292 NHs that were contacted responded to the survey as of May 5. A total of 52% of the NHs responded within 2 weeks from the first email. A response rate higher than 40% was achieved in some of the regions with a high number of COVID‐19 cases (Table 1). However, a negative association was observed between the response rate and the attack rate per region, even if not statistically significant (Spearman's rho = ‐0.21, *p* = 0.344). Two of the 21 regions did not participate in the survey.

**TABLE 1:** Distribution and description of facilities (response rate, number of participating nursing homes, residents, number of beds, and average number of beds per unit of staff), overall and by region

Italian regions	Response rate	Nursing homes	Number of residents^a^	Beds per facility, median [range]	Beds‐to‐staff ratio,^b^ mean ± sd
Piedmont	41.0	249	17,186	60 [10–288]	2.3 ± 0.7
Valle D'Aosta	0.0	0	‐		‐
Lombardy	43.1	292	27,657	80 [20–448]	2.2 ± 1.5
AP Bolzano	10.8	4	425	88 [81–204]	1.5 ± 0.5
AP Trento	29.4	15	1201	67 [60–187]	1.4 ± 0.2
Veneto	28.5	148	17,902	104.5 [16–667]	1.9 ± 1.4
Friuli Venezia Giulia	55.7	39	3636	68 [20–368]	2.1 ± 0.7
Liguria	17.2	20	1573	62.5 [24–240]	2.4 ± 0.8
Emilia Romagna	46.0	128	8200	58.5 [18–187]	1.8 ± 0.7
Tuscany	62.7	200	9607	42 [12–205]	1.8 ± 1.2
Umbria	38.1	16	730	35.5 [20–90]	1.6 ± 0.2
Marche	90.8	36	1384	31.5 [15–129]	1.4 ± 0.4
Latium	41.1	79	4597	56 [9–160]	2.1 ± 0.7
Abruzzo	49.0	8	447	40 [20–113]	1.9 ± 0.7
Molise	66.7	4	233	57 [40–110]	2.6 ± 0.8
Campania	13.2	16	642	40 [20–76]	2.0 ± 0.5
Apulia	57.4	35	2088	60 [9–125]	1.9 ± 0.7
Basilicata	0.0	0	‐		‐
Calabria	45.0	36	1557	40 [8–100]	1.6 ± 0.4
Sicily	61.5	24	1132	40 [10–94]	1.5 ± 0.4
Sardinia	43.8	7	609	72 [42–189]	1.5 ± 0.3
					‐
North‐West	40.0	561	46,416	70 [10–448]	2.2 ± 1.2
North‐Est	34.9	334	31,364	71 [16–667]	1.8 ± 1.1
Center	55.8	331	16,318	40 [9–205]	1.8 ± 1.0
South and Islands	36.8	130	6708	44 [8–189]	1.8 ± 0.6
Overall	41.2	1356	100,806	60 [8–667]	2 ± 1.1

Abbreviation: AP, Autonomous Province.

^a^ Number of residents = residents present at February 1 and newly admitted since March 1.

^b^ Staff includes medical doctors, nurses and health care social workers.

All questionnaires were considered as completed, as the proportion of answers was higher than 93% for all questions and 98% for crucial questions. The number of residents was missing for seven NHs, therefore these data were imputed using the number of beds.

Overall, a total of 100,806 residents were living in the interviewed NHs (Table 1).

### 3.2 Characteristics of the facilities

This survey included either public structures or structures providing services both privately and within the NHS. A median of 60 beds (range 8–667) per facility was reported, with a wide variability between regions. When considering the number of HCSW, the NHs reported a median of 32 workers per facility, with a mean of two beds per HCSW in each facility (Table 1).

### 3.3 Spreading of COVID‐19 among NHs residents and member of staff

Overall, 9154 residents died from February 1 to the time when the questionnaire was completed (from March 25 to May 5). Most of the deaths occurred between March 16 and March 31 (33.8%). A total of 680 deceased residents (7.4%) had a laboratory‐confirmed diagnosis of COVID‐19, and 3092 deceased residents (33.8%) were reported as having flu‐like symptoms, thus accounting for a cumulative incidence of 0.7 per 100 residents and 3.1%, respectively. A wide variability among regions was observed (Table 2). A positive correlation was observed between the cumulative incidence of death within the facility and the attack rate within the corresponding Region when at the date the questionnaire was completed (Spearman's rho = 0.336, *p* < 0.001).

**TABLE 2:** Deaths due to any cause, related to SARS‐Cov‐2 (laboratory‐confirmed) and related to patients with flu‐like symptoms occurred between February 1 and May 5, overall and by region

	Number of deaths			Cumulative incidence, per 100 in‐residents		
Italian regions	Any cause	SARS‐Cov‐2+, n (%)	Flu‐like symptoms, *n* (%)	Any cause	SARS‐Cov‐2+	Flu‐like symptoms
Piedmont	1658	161 (9.7)	410 (24.7)	9.6	0.9	2.4
Lombardy	3793	281 (7.4)	1807 (47.6)	13.7	1.0	6.5
AP Bolzano	28	3 (10.7)	10 (35.7)	6.6	0.7	2.4
AP Trento	99	33 (33.3)	45 (45.5)	8.2	2.7	3.7
Veneto	1136	38 (3.3)	180 (15.8)	6.3	0.2	1.0
Friuli Venezia Giulia	222	6 (2.7)	41 (18.5)	6.1	0.2	1.1
Liguria	136	20 (14.7)	34 (25)	8.6	1.3	2.2
Emilia Romagna	639	81 (12.7)	265 (41.5)	7.8	1.0	3.2
Tuscany	640	36 (5.6)	154 (24.1)	6.7	0.4	1.6
Umbria	38	0 (0)	11 (28.9)	5.2	0.0	1.5
Marche	160	13 (8.1)	59 (36.9)	11.6	0.9	4.3
Latium	158	1 (0.6)	28 (17.7)	3.4	0.0	0.6
Abruzzo	47	1 (2.1)	0 (0)	10.5	0.2	0.0
Molise	24	0 (0)	2 (8.3)	10.3	0.0	0.9
Campania	50	6 (12)	13 (26)	7.8	0.9	2.0
Apulia	111	0 (0)	4 (3.6)	5.3	0.0	0.2
Calabria	75	0 (0)	1 (1.3)	4.8	0.0	0.1
Sicily	73	0 (0)	11 (15.1)	6.4	0.0	1.0
Sardinia	67	0 (0)	17 (25.4)	11.0	0.0	2.8
*North‐West*	*5587*	*462 (8.3)*	*2251 (40.3)*	*12.0*	*1.0*	*4.8*
*North‐Est*	*2124*	*161 (7.6)*	*541 (25.5)*	*6.8*	*0.5*	*1.7*
*Center*	*996*	*50 (5)*	*252 (25.3)*	*6.1*	*0.3*	*1.5*
*South and Islands*	*447*	*7 (1.6)*	*48 (10.7)*	*6.7*	*0.1*	*0.7*
Overall	9154	680 (7.4)	3092 (33.8)	9.1	0.7	3.1

Abbreviations: AP, Autonomous Province; SARS‐CoV‐2, severe acute respiratory syndrome coronavirus 2.

Overall, 5292 residents were hospitalized in the 1342 facilities that provided this information (Table 3).

**TABLE 3:** Hospitalization due to any cause, related to residents with laboratory‐confirmed COVID‐19 and residents with flu‐like symptoms, overall and by region

	Hospitalizations					
	any cause		SARS‐CoV‐2^+^		flu‐like symptoms	
Italian regions	N	mean number per facility	*N* (%)	Rates per 100 residents^§^	*N* (%)	Rates per 100 residents^§^
Piedmont	1048	4.2	362 (34.5)	2.1	496 (47.3)	2.9
Lombardy	719	2.5	198 (27.5)	0.7	370 (51.5)	1.4
AP Bolzano	27	6.8	5 (18.5)	1.2	6 (22.2)	1.4
AP Trento	53	3.5	4 (7.5)	0.3	38 (71.7)	3.2
Veneto	933	6.3	65 (7)	0.4	226 (24.2)	1.3
Friuli Venezia Giulia	341	9.0	18 (5.3)	0.5	114 (33.4)	3.2
Liguria	111	5.6	15 (13.5)	1.0	38 (34.2)	2.4
Emilia Romagna	604	4.7	136 (22.5)	1.7	278 (46)	3.4
Tuscany	732	3.7	87 (11.9)	0.9	247 (33.7)	2.6
Umbria	33	2.1	1 (3)	0.1	19 (57.6)	2.6
Marche	137	3.9	30 (21.9)	2.2	60 (43.8)	4.4
Latium	212	2.7	14 (6.6)	0.3	48 (22.6)	1.1
Abruzzo	33	4.1	0 (0)	0.0	6 (18.2)	1.3
Molise	9	2.3	0 (0)	0.0	5 (55.6)	2.1
Campania	65	4.1	30 (46.2)	4.7	18 (27.7)	2.8
Apulia	68	2.0	0 (0)	0.0	9 (13.2)	0.4
Calabria	30	0.8	0 (0)	0.0	5 (16.7)	0.3
Sicily	92	3.8	0 (0)	0.0	27 (29.3)	2.4
Sardinia	45	6.4	0 (0)	0.0	11 (24.4)	1.8
*North‐West*	1878	3.4	575 (30.6)	1.3	904 (48.1)	2.0
*North‐Est*	1958	5.9	228 (11.6)	0.7	662 (33.8)	2.1
*Center*	1114	3.4	132 (11.8)	0.8	374 (33.6)	2.3
*South and Islands*	342	2.7	30 (8.8)	0.4	81 (23.7)	1.2
Overall	5292	3.9	965 (18.2)	1.0	2021 (38.2)	2.0

Abbreviations: COVID‐2019, Coronavirus disease 2019; SARS‐CoV‐2, severe acute respiratory syndrome coronavirus 2.

^§^Residents = people living in Nursing Home at February 1 and newly admitted since March 1.

A total of 161 NHs (12.0%) out of the 1337 that answered the question, had SARS‐CoV‐2 positive residents at the date they completed the questionnaire. Facilities with a COVID‐19 outbreak were located in 12 of the 19 regions participating in the survey and had a mean number of 18.7 positive cases per 100 residents, ranging from 0.5 and 86.9 per 100 residents. The highest number of positive residents was reported in Piedmont, with 826 positive residents in 40 of the 243 local NHs (16.5% of the NHs with a mean number of 27.2 per 100 residents). Starting March 29, a further question on the number of residents with flu‐like symptoms was included. Therefore, only 1088 facilities (80.2% of the total) provided this information. A total of 381 NHs had at least one resident with flu‐like symptoms, that is the 35.0% of the NHs across all the 19 Regions, with a mean number of 9.5 persons with flu‐like symptoms per 100 residents (ranging from 0.3 to 100). Lombardy was the Region with the highest number of cases, with the 57.8% of NHs reporting residents with flu‐like symptoms.

Moreover, 278 NHs out of the 1320 that reported the information, had SARS‐CoV‐2 positive staff members (21.1%), with the majority of them being in northern Italy.

Overall, when considering the number of SARS‐CoV‐2 positive residents or staff members deceased or hospitalized, outbreaks occurred in 385 NHs out of 1326 (29%). When considering also flu‐like symptoms, the outbreaks involved 67.7% of the included NHs (909 out of 1343).

### 3.4 Infection prevention and control

The management of residents with COVID‐19 (suspected or laboratory‐confirmed) manly relied on physicians/HCSW within the facility (41.4%) and on general practitioners (26.7%). Written guidelines for the appropriate management of residents with COVID‐19 were available in 92.9% of the NHs, but 59.4% did not receive any ad hoc consultation for neither the management of patients nor IPC. No specific training for COVID‐19 IPC was provided to HCSWs in 35.1% of the included NHs, while 93.3% of the NHs provided some training for staff members on the appropriate use of PPE. Moreover, 91.5% of the NHs provided information and raised awareness on COVID‐19 among residents.

All the NHs, except for one, suspended visits from relatives/caregivers to the residents with almost all of them (99.5%) providing alternative means for communication.

**FIGURE 2:**
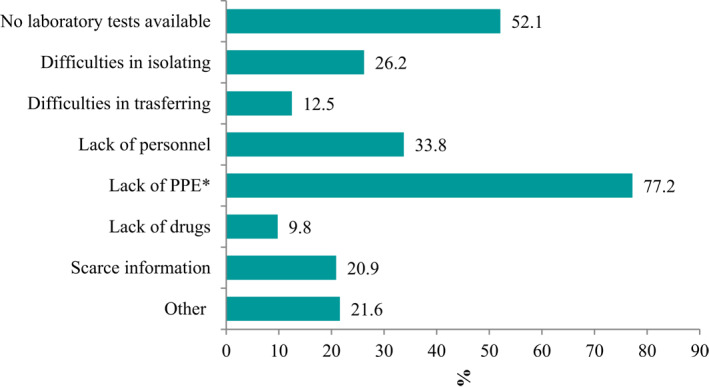
Difficulties faced during the epidemic

As for the main critical issues faced during the epidemic, 77.2% out of the 1259 NHs that answered this question, reported a lack of PPE, 52.1% were not able to obtain laboratory tests (data available starting April 9, thus referring to 541 NHs; Figure 2).

Moreover, 7.7% of the NHs reported that they were not able to isolate residents with suspected or confirmed COVID‐19. The majority of respondents (47.8%) reported they were able to isolate patients in a single room, 29.9% could organize a room with only cases of COVID‐19, only 3.6% were able to transfer patients to a dedicated facility, and 11.0% reported other methods for isolation, mostly combinations of the listed options.

Most NHs (79.1%) reported to monitor the temperature among residents and staff members twice a day. A total of 1045 NHs reported data on influenza vaccination, with an overall 86.1% coverage, and 21.3% NHs reporting a full coverage.

### 3.5 Factors associated with the spreading of COVID‐19

Univariate and multivariate logistic models were performed to investigate the possible association between some critical aspects and characteristics of the NHs and the likelihood of being no COVID‐19 free NHs (laboratory‐confirmed). The multivariate analysis showed a negative association between lack of PPE and COVID‐19 outbreak status of the investigated NHs. This association varies along with the considered time period, with a strong negative association within the first 3 weeks (OR = 0.45, *p* < 0.001), and no association within the following 3 weeks (OR = 0.88, ns). The lack of personnel (OR = 3.22, *p* < 0.001), the difficulty in transferring to hospital or other facility COVID‐19 patients (OR = 4.67, *p* < 0.001) and isolating (OR = 1.98, *p* < 0.001) residents, a size of the facility higher than the median of 60 beds (OR = 1.50, *p* = 0.013), and the geographic area (OR = 7.6, 6.6, 3.3 for respectively N‐W, N‐E, Center vs. South) were all positively associated to the no COVID‐19 free status (Table 4).

**TABLE 4:** Crude and adjusted ORs by univariate and multivariate logistic model, estimating the association with no COVID‐19 free status in Nursing Homes

	Crude OR			Adjusted OR^a^		
Variables	OR_cr_	*p‐value*	95% CI	OR_adj_ ^a^	*p‐value*	95% CI
Lack of PPE (Y vs. N)						
In the first 3 weeks	0.58	*0.003*	(0.41–0.83)	0.45	< *0.001*	(0.29–0.68)
After 3 weeks	1.10	*0.694*	(0.66–1.87)	0.88	*0.672*	(0.47–1.62)
Lack of laboratory tests^b^ (Y vs. N)	1.62	*0.009*	(1.13–2.34)	0.68	*0.118*	(0.41–1.10)
Scarce information (Y vs. N)	1.53	*0.003*	(1.15–2.05)	1.00	*0.995*	(0.69–1.44)
Lack of personnel (Y vs. N)	4.57	< *0.001*	(3.52–5.92)	3.22	< *0.001*	(2.38–4.36)
Difficulty in transferring (Y vs. N)	10.57	< *0.001*	(7.12–15.7)	4.66	< *0.001*	(2.98–7.31)
Difficulty in isolating (Y vs. N)	3.31	< *0.001*	(2.54–4.33)	1.97	< *0.001*	(142–2.73)
Lack of drugs (Y vs. N)	2.76	< *0.001*	(1.88–4.04)	1.54	*0.072*	(0.96–2.46)
Median number of beds (upper vs. below 60 beds)	1.98	< *0.001*	(1.54–2.53)	1.50	*0.013*	(1.09–2.07)
Beds‐to‐staff ratio	1.20	*0.001*	(1.07–1.33)	1.07	*0.328*	(0.93–1.24)
Geographic region						
North‐West	15.65	< *0.001*	(6.78–36.14)	7.60	< *0.001*	(2.93–19.7)
North‐Est	7.56	< *0.001*	(3.22–17.78)	6.61	< *0.001*	(2.51–17.43)
Center	3.88	*0.002*	(1.62–9.29)	3.30	*0.018*	(1.23–8.90)
South	1			1		

Abbreviations: COVID‐2019, Coronavirus disease 2019; PPE, personal protective equipment.

^a^ Adjusted for all the variables listed in the table, except for lack of laboratory tests. The interaction term between lack of PPE and period of response (≤3 or >3 weeks) was added in the multivariate model since it was significant at 5% level in the univariate analysis.

^b^ This information was gathered in a second wave of the survey, therefore the OR is referred to a model performed in a subset of data collected since April 8, that is starting week 3 (*n* = 598).

The sensitivity analysis performed including influenza‐like symptoms in the definition of no COVID‐19 free NH substantially confirmed the results (Table S1).

## 4 DISCUSSION

To our knowledge, this is the first national survey carried out among nursing home conducted during the COVID‐19 pandemics.

The mortality profile in the Italian NHs was influenced by the spreading of the epidemics among the Italian regions. In particular, a higher mortality rate was observed in the northern regions of Italy.

Moreover, in the Center, South and Islands of Italy, the observed outbreaks were mainly delimited in the areas where NHs were located.^23^ However Italy experienced a strong heterogeneity of COVID‐19 outbreaks between regions and the reason is probably that the northern regions are geographic settings more connected with other countries in terms of movement of people and goods.

This survey also aimed at highlighting the potential underestimation of the mortality during the pandemic, by reporting the proportion of deceased in each NH that had flu‐like symptoms and might not have performed a swab test for COVID‐19. Official statistics across all countries reported the number of deceased with laboratory‐confirmed COVID‐19, but there is most likely a relevant proportion of deceased who had flu‐like symptoms but did not undergo a nasopharyngeal swab. A document from the INH of Statistics and the INIH, estimated a 94.9 % increase in all‐cause mortality in March 2020 in the North of Italy, a 9.1% increase in the Center, and a 2.0% increase in the South and Islands compared to the mean mortality in same month in the previous five years.^24^

Some critical issues characterized NHs with COVID‐19 outbreak compared to ones without an outbreak, such as lack of personnel, difficulty in transferring to hospital or other facility patients or isolating them in a single room, lack of medications and impossibility to perform swab tests. Moreover, the NHs with a higher number of beds were probably at a higher risk to develop an outbreak (Table 4).

The association between lack of PPE and status of the NHs was different depending on whether NHs responded to the survey within the first 3 weeks of the survey or after 3 weeks (Table 4). This is probably due to when the questionnaire was completed, considering that the number of facilities with an active outbreak increased over the 6 weeks of the survey, while the absence of PPE decreased over time. However, due to the nature of the survey, the association between absence of PPE and status of the included NHs cannot be easily investigated.

Based on the needs emerged during the survey, the INIH organized a telephone counseling for the most critical facilities, a distance training course for NHs HCSW, a series of webinars on ICP practices for the management of NHs residents, and produced training and information materials on IPC.

The only national study on this issue using current information flows was conducted in the US NHs.^25^ Of 9395 NHs, 2949 (31.4%) had a documented COVID‐19 case.^25^ Larger facility size, urban location, greater percentage of African American residents, nonchain status, and state were significantly (*p* < 0.05) related to increased probability of having a COVID‐19 case.^25^

At a regional level, in Ontario (Canada), on 627 log‐term facilities 272 (43.4%) were identified as having either confirmed or suspected COVID‐19 infection in residents or staff.^26^

The main strength of our study is to report the results of a survey carried during the most critical phase of the pandemic, while its limits are mainly due to the lack of data on individuals (residents and HCSW) and the 41% response rate.

The Italian Dementia Observatory provides only the list of NHs presents in the Italian regions and does not collect characteristics of the facilities preventing from carrying out an analysis between the NHs who participated in the survey and those who did not participate.

However, we attempted to assess the nonresponse bias in our survey by observing an inverse correlation between the response rate to the survey within the different regions and the corresponding infection attack rate. Moreover, we identified from news reports the information on 73 NHs that had outbreaks of COVID‐19. Of these, only 20 (27%) had responded to our survey. This shows that NHs which had problems during the pandemics might have not responded to our survey, and thus the results of this study might report an underestimation of what happened in all Italian NHs.

We believe that this survey might help to highlight the potential underestimation of the mortality due to Covid‐19, and the critical issues that have affected the NHs across all the countries^6^ ^,^ ^8^ ^,^ ^12^ with the objective of protecting one of the most vulnerable group of population.

### 4.1 Lessons learned so far and implementation of active surveillance systems

Measures adopted in the first phase of COVID‐19 outbreak in Italy contributed significantly to the flattening of the epidemic curve with reduction of new cases and consequent lightening of the care response borne by the health service. However, human lives lost, especially among elderly residents living in NHs due to SARS‐CoV‐2 exposed major flaws in health care system. Currently, the consolidation of a new phase, characterized by initiatives to stop the lockdown and their progressive extension, it can only take place where it is insured close monitoring of virus transmission on the national territory. Moreover, preparedness of health care system, contact tracing, monitoring of people in quarantine, prompt nasopharyngeal swab tests, link between primary care and hospitalization, as well as a timely supply of information flows through the insertion of routine data or implemented information systems ad hoc for the ongoing emergency are key factors for Italy to enter in phase II. Therefore, according to the Decree of the Italian Prime Minister of 26 April 2020 for the monitoring purposes some indicators (including one for nursing home) have been defined with threshold and alert values to be monitored, through national coordinated surveillance systems, in order to obtain aggregated national, regional and local data. Monitoring includes the following indicators: (a) process indicators on monitoring capacity; (b) process indicators on the capacity of diagnostic assessment, investigation, and contact tracing; and (c) result indicators related to transmission stability and resiliency of health services. Therefore, through these indicators, a risk classification will be updated weekly for each region/autonomous province. The Minister of Health, through a control room, will involve the National INIH and all regions/P.A. to collect all information necessary for an updated risk classification to avoid an uncontrolled and unmanageable transmission of SARS‐CoV‐2. To build an efficient research and contact‐tracing system, human resources will have to be rapidly available and adequately trained.

## AUTHOR CONTRIBUTIONS

Nicola Vanacore, Graziano Onder, Fortunato D’Ancona, Marco Canevelli, and Gilda Losito conceived the study. Flavia L. Lombardo, Flavia Mayer, Antonio Bella, and Paola Piscopo worked on statistical aspects of the study. Ilaria Bacigalupo, Emanuela Salvi, Eleonora Lacorte, Paola Piscopo, Antonio Ancidoni, Giulia Remoli, and Guido Bellomo were involved in the organization of the study. The Italian National INIH Nursing Home Study Group performed the study. All authors interpreted the results, contributed to writing the Article, and approved the final version for submission. List of the Italian National Institute of Health Nursing Home Study Group co‐authors: Antonio Ancidoni, Ilaria Bacigalupo, Guido Bellomo, Luigi Bertinato, Marco Canevelli, Patrizia Carbonari, Maria Grazia Carella, Annamaria Confaloni, Alessio Crestini, Fortunato D'Ancona, Carla Faralli, Simone Fiaccavento, Silvia Francisci, Flavia L. Lombardo, Eleonora Lacorte, Cinzia Lo Noce, Paola Luzi, Tania Lopez, Flavia Mayer, Maria Masocco, Monica Mazzola, Graziano Onder, Ilaria Palazzesi, Luana Penna, Daniela Pierannunzio, Paola Piscopo, Maria Cristina Porrello, Giulia Remoli, Emanuela Salvi, Giulia Scaravelli, Andrea Siddu, Sabrina Sipone, Lucia Speziale, Andrea Tavilla, Nicola Vanacore (Italian National Institute of Health). Mauro Palma (President) and Gilda Losito (Head of Unit—Deprivation of liberty and Health protection), Italian National Guarantor for the rights of persons detained or deprived of liberty. Gianluca Pucciarelli (Dipartimento di Biomedicina e Prevenzione‐Università di Tor Vergata), Daniela Accorgi (UsL Centro Toscana), Catia Bedosti (Ausl Imola‐ Emilia Romagna), Gabriella Carraro (Aulss two Veneto) Maria Mongardi (Dipartimento di Malattie Infettive—Università di Verona). Gianluca Ferrari (Area Comunicazione e Informatica srl).

## CONFLICT OF INTEREST

We declare no competing interests.

## Supporting information

Supplementary Material

## Data Availability

The data that support the findings of this study are available on request from the corresponding author.
