# Supplementary material for: The Italian national survey on Coronavirus disease 2019 epidemic spread in nursing homes
Source: Int J Geriatr Psychiatry. 2021 Jan 2;36(6):873–82. doi: 10.1002/gps.5487 (PMC8247061; doi:10.1002/gps.5487)
Supplement: Supplementary file 1 — Supplementary Material [file GPS-36-873-s001.docx]

***Additional/Supplementary information***

**Supplementary Table S1 – Crude and adjusted ORs by univariate and multivariate logistic model, estimating the association with no COVID-19 free status including also** **flu-like symptoms**

|  | **Crude OR** | | |  | **Adjusted OR^a^** | | |
| --- | --- | --- | --- | --- | --- | --- | --- |
| **Variables** | **OR_cr_** | ***p-value*** | **95%CI** |  | **OR_adj_^a^** | ***p-value*** | **95%CI** |
| **Lack of PPE** (Y vs N) |  |  |  |  |  |  |  |
| in the first 3 weeks | 0.86 | *0.395* | (0.60-1.22) |  | 0.74 | *0.140* | (0.50-1.10) |
| after 3 weeks | 1.23 | *0.483* | (0.69-2.20) |  | 1.07 | *0.834* | (0.57-2.01) |
| **Lack of laboratory tests ^b^** (Y vs N) | 1.57 | *0.016* | (1.09-2.26) |  | 1.27 | *0.286* | (0.82-2.00) |
| **Scarce information** (Y vs N) | 1.15 | *0.359* | (0.85-1.55) |  | 0.95 | *0.751* | (0.67-1.33) |
| **Lack of personnel** (Y vs N) | 3.03 | *<0.001* | (2.27-4.05) |  | 2.15 | *<0.001* | (1.57-2.95) |
| **Difficulties in transferring** (Y vs N) | 9.89 | *<0.001* | (4.80-20.36) |  | 5.44 | *<0.001* | (2.57-11.53) |
| **Difficulties in isolating** (Y vs N) | 2.18 | *<0.001* | (1.61-2.96) |  | 1.43 | *0.039* | (1.02-2.00) |
| **Lack of drugs** (Y vs N) | 3.25 | *<0.001* | (1.89-5.57) |  | 2.00 | *0.018* | (1.13-3.58) |
| **Median number of beds** (upper vs below 60) | 2.65 | *<0.001* | (2.09-3.35) |  | 2.12 | *<0.001* | (1.60-2.79) |
| **Beds-to-staff ratio** | 1.07 | *0.241* | (0.95-1.20) |  | 1.01 | *0.827* | (0.88-1.17) |
| **Geographic region** |  |  |  |  |  |  |  |
| North-West | 4.55 | *<0.001* | (3.04-6.82) |  | 2.56 | *<0.001* | (1.61-4.07) |
| North-Est | 5.38 | *<0.001* | (3.47-8.35) |  | 4.22 | *<0.001* | (2.59-6.90) |
| Centre | 2.23 | *<0.001* | (1.47-3.40) |  | 2.45 | *<0.001* | (1.52-3.96) |
| South | 1 |  |  |  | 1 |  |  |
|  |  |  |  |  |  |  |  |

^a Adjusted for all the variables listed in the table, except for lack of laboratory test. The interaction term between lack of PPE and period of response (≤3 or >3 weeks) was added in the multivariate model since it was significant at 5%level in the univariate analysis.^

^b This information was gathered in a second wave of the survey, therefore the OR is referred to a model performed in a subset of data collected since April 8 i.e. starting week 3 (n=598).^

^Note. No COVID-19 free status defined as the presence of flu-like symptoms among deceased and hospitalized residents or residents living in the facility at the date of completion the questionnaire.^

**English version of the Questionnaire**

**
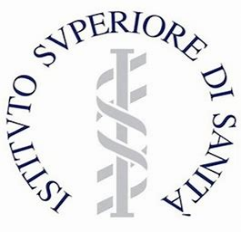
**

**QUESTIONNAIRE ON COVID-19 INFECTION IN NURSING HOMES**

**Name of the structure**

**Type of structure:**

**options:** Providing services within the NHS [ ]

Providing services both privately and within the NHS [ ]

Providing services only privately [ ]

*Other* [specify: ]

**Business name:**

**Address:**

**City:**

**Province/District:**

**Region:**

**Phone number (you can provide more than one number):**

**E-mail address:**

**Who is completing the questionnaire:**

**First name and last name:**

**Role within the structure:**

**E-mail address:**

**Data in which the questionnaire was completed:**

**1. What is the total number of healthcare and social workers (HCSW) who are currently operating in the structure?**

- Physicians, specify the total number: [ ]

- Nurses, specify the total number: [ ]

- Other HCSW, specify the total number: [ ]

*other members of the staff that could have been in close contact with the patients (e.g. educators, entertainers, psychologists, etc.):*

*-* Social workers, number: [ ]

*-* Physiotherapists/therapists/rehabilitators, number: [ ]

- Psychologists, number: [ ]

- Educators/entertainers, number: [ ]

- Other, specify type: number: [ ]

**2. How many beds are available in the facility?**

Number:  [ ]

**3. How many residents were in the facility on February 1?**

Number:  [ ]

**4. How many residents have died since February 1?** [ ]

Specify the number for each time period:

**February 1-15:** [ ]

**February 16-29:** [ ]

**March 1-15:** [ ]

**March 16-31:** [ ]

**April 1-15:** [ ]

**April 16 -30:** [ ]

**5. How many residents who died in the facility since February 1 had a positive swab test for COVID-19?**

Number:  [ ]

**6. Overall, how many residents who died in the facility since February 1 had influenza-like symptoms, respiratory symptoms (such as fever, chough or dyspnea) or pneumonia (irrespective of their having undergone a test for COVID-19)?**

 Number:  [ ]

**7. How many residents have been hospitalised since February 1?**

Number:  [ ]

**8. How many residents who were hospitalised since February 1 had a positive swab test for COVID-19?**

  Number:  [ ]

**9.** **How many residents who were hospitalised since February 1 had influenza-like symptoms, respiratory symptoms (such as fever, chough or dyspnea) or pneumonia (irrespective of their having undergone a test for COVID-19)?**

Number: [ ]

**10. How many residents were newly admitted to your facility since March 1?**

Number: [ ]

**11. How many patients are currently present in your facility who either have a positive swab test for COVID-19 or influenza-like symptoms/pneumonia?**

- positive swab test for COVID-19, number: [ ]

- influenza-like symptoms/pneumonia, (irrespective of having undergone a test for COVID-19, number: [ ]

**12. What are the main difficulties you are facing during the coronavirus epidemics?**

(more that one option is allowed, mark with an X)

a.     Lack of information provided on the procedures to be carried out to contain the infection [ ]

b.    Shortage of drugs and medications

c.     Lack of personal protective equipment (PPE) [ ]

d.    Absence of personnel [ ]

e.     Difficulty in transferring the residents with COVID-19 to hospitals [ ]

f.      Difficulties in isolating the residents with COVID-19 [ ]

g. Impossibility of having suspect cases undergo a swab test for Sars-Cov-2

f.     Other (specify):

**13.  In agreement with the legislation (DPCM) issued March 8, 2020, did you suspend all visits from relatives/caregivers to the residents?**

- No
- If yes, specify since when the visits were suspended: mm/dd/yyyy
- If visits were allowed, specify any exceptions (e.g. end of life, other):

**14. Did you provide alternative means for communication between residents and their relatives/caregivers?**

- NO [ ]

- YES [ ]

If yes, specify:

- since when the visits were suspended: mm/dd/yyyy

- which means were provided:

- videocalls No [ ] Yes[ ]

- phone calls No [ ] Yes [ ]

- other (specify):

**15. Did any member of the staff in the facility have a positive swab test for COVID-19?**

- NO [ ]

- YES [ ]

**16. Do you have a written plan/procedure for the management of residents with suspected or confirmed COVID-19?**

- NO [ ]

- YES [ ]

**17. Did you receive an ad hoc consultation for the clinical management and/or prevention and control of COVID-19?**

- NO [ ]
- YES [ ] (specify the type of consultation):

**18. Who is in charge of managing the residents with suspected or confirmed COVID-19?**

 (mark with an X)

a.     GPs [ ]

b.    Physicians within the facility [ ]

c.     External consultants [ ]

d.    Other (specify): [ ]

**19. Are you able to isolate residents in case of suspected or confirmed Sars-Cov-2 infection?**

 (mark with an X)

- No[ ]
- Yes (private room) [ ]
- Yes (room with only residents with COVID-19) [ ]
- Yes (transferal to another facility) [ ]
- Yes (other specify): [ ]

**20. Does the facility register and monitor all applied physical restraint measures?**

- NO [ ]
- YES [ ]

**21. How many physical restraint measures were applied since February 1 for the management of the residents?**

- Number: [ ]

**22. Have you noticed an increase in the prescription of psychotropic medications (benzodiazepines, antidepressant or antipsychotic agents) since February 1?**

- NO [ ]

- YES [ ]

If YES, specify for what type of medication:

- benzodiazepines [ ]

- antidepressant drugs [ ]

- antipsychotic drugs [ ]

**23. Have you noticed any adverse event (e.g. accidents, confrontations, falls…) since February 1?**

- NO [ ]

- YES [ ]

If yes, specify the number of:

- Adverse events among the staff: [ n° ]
- Adverse events among the residents: [ n° ]
- Adverse events involving both the staff and residents: [ n° ]

**24. Have you implemented a training program for healthcare and social workers including also specific practical activities on COVID-19 (NIH courses, videos…)?**

- NO[ ]
- YES [ ]

**25*.* Have you implemented a training course for healthcare and social workers on the appropriate use of PPE?**

- NO [ ]
- YES [ ]

**26. Did you take any initiative to share information and raise awareness among residents on the prevention and control of COVID-19?**

- NO [ ]
- YES [ ]

**27. Are there hand sanitizer dispensers available for staff to use?**

- NO [ ]
- YES [ ]

**28. Do you measure the temperature twice a day among both residents and staff members?**

- NO [ ]
- YES [ ]

**29. What is the influenza vaccine coverage of the residents in the facility?**

- Specify the percentage: [ ]

**Thank you for your cooperation**
